# Supplementary material for: Perinatal complications of the maternal–Fetal dyad in primiparous women subjected to vaginal delivery versus elective cesarean section: A retrospective study of clinical results associated with bioethical precepts
Source: PLoS One. 2023 Oct 17;18(10):e0292846. doi: 10.1371/journal.pone.0292846 (PMC10581477; doi:10.1371/journal.pone.0292846)
Supplement: Attachment — Submitted filename: PLOS ONE-AUTONOMY IN CHOOSING THE MODE OF DELIVERY.docx [file pone.0292846.s001.docx]

PLOS ONE

Autonomy of pregnant women in choosing the mode of delivery: A retrospective study

--Manuscript Draft--

Manuscript Number: PONE-D-22-30934

Article Type: Research Article

Full Title: Autonomy of pregnant women in choosing the mode of delivery: A retrospective study

Short Title: Autonomy of pregnant women in choosing the mode of delivery

Corresponding Author: Carlos Henrique Mascarenhas Silva, M.D

Mater Dei Hospital

Belo Horizonte, BRAZIL

Keywords: Bioethics, obstetrics

Abstract: Obstetrics is undergoing transformation and committing to ensuring the autonomy of

pregnant women in decisions related to birth based on scientific information. Vaginal

delivery results from the physiological process of birth, but medicine has evolved to

include obstetric surgeries that are safe and have few perioperative complications,

especially when cesarean section is performed from 39 weeks of gestational age.

Thus, the question is whether the clinician should continue to interfere with the freedom

of the pregnant woman to choose the mode of delivery when trying to persuade her to

choose vaginal delivery for the birth of her child. To analyze the perinatal complications

of the maternal–fetal binomial of primiparous women subjected to vaginal delivery

versus elective cesarean section. In total, 2,507 deliveries, including 1,807 (72.1%)

vaginal deliveries and 700 (27.9%) cesarean deliveries, were analyzed between 2017

and 2020. There was no difference in maternal readmission, death, admission to the

intensive care unit, Apgar score <7 in the 5th minute of life, maternal blood transfusion

or comorbidities of mothers or newborns between the types of delivery. The elective

cesarean section showed less need for therapeutic uterotonics. It was observed that

elective cesarean section in primigravidae does not present a higher risk of

complications than vaginal delivery. This guarantees, therefore, the autonomy and right

of the individual to choose the mode of delivery.

Order of Authors: Carlos Henrique Mascarenhas Silva

Cláudia Lourdes Soares Laranjeira

Carolina Soares Barros de Melo, Ph.D., M.D.

Lorena Ventura Brandão

Gabriela Costa Oliveira

Augusto Henrique Fulgêncio Brandão

Rui Nunes

Additional Information:

Question Response

Financial Disclosure

Enter a financial disclosure statement that

describes the sources of funding for the

work included in this submission. Review

the submission guidelines for detailed

requirements. View published research

articles from PLOS ONE for specific

examples.

This statement is required for submission

The author(s) received no specific funding for this work. The funders had no role in

study design, data collection and analysis, decision to publish, or preparation of the

manuscript.

*Powered by Editorial Manager® and ProduXion Manager® from Aries Systems Corporation*

and will appear in the published article if

the submission is accepted. Please make

sure it is accurate.

Unfunded studies

Enter: The author(s) received no specific

funding for this work.

Funded studies

Enter a statement with the following details:

Initials of the authors who received each

award

•

• Grant numbers awarded to each author

• The full name of each funder

• URL of each funder website

Did the sponsors or funders play any role in

the study design, data collection and

analysis, decision to publish, or preparation

of the manuscript?

•

NO - Include this sentence at the end of

your statement: The funders had no role in

study design, data collection and analysis,

decision to publish, or preparation of the

manuscript.

•

• YES - Specify the role(s) played.

* typeset

Competing Interests

Use the instructions below to enter a

competing interest statement for this

submission. On behalf of all authors,

disclose any competing interests that

could be perceived to bias this

work—acknowledging all financial support

and any other relevant financial or nonfinancial

competing interests.

This statement is required for submission

and will appear in the published article if

the submission is accepted. Please make

sure it is accurate and that any funding

sources listed in your Funding Information

later in the submission form are also

declared in your Financial Disclosure

statement.

View published research articles from

PLOS ONE for specific examples.

The authors have declared that no competing interests exist. .

*Powered by Editorial Manager® and ProduXion Manager® from Aries Systems Corporation*

NO authors have competing interests

Enter: The authors have declared that no

competing interests exist.

Authors with competing interests

Enter competing interest details beginning

with this statement:

I have read the journal's policy and the

authors of this manuscript have the following

competing interests: [insert competing

interests here]

* typeset

Ethics Statement

Enter an ethics statement for this

submission. This statement is required if

the study involved:

• Human participants

• Human specimens or tissue

• Vertebrate animals or cephalopods

• Vertebrate embryos or tissues

• Field research

Write "N/A" if the submission does not

require an ethics statement.

General guidance is provided below.

Consult the submission guidelines for

detailed instructions. Make sure that all

information entered here is included in the

Methods section of the manuscript.

The name of the ethics committee that approved the study:Mater Dei Health Network,

Brazil / seem (4.400.669)

Approval number 36135120.5.0000.5128

*Powered by Editorial Manager® and ProduXion Manager® from Aries Systems Corporation*

Format for specific study types

Human Subject Research (involving human

participants and/or tissue)

Give the name of the institutional review

board or ethics committee that approved the

study

•

Include the approval number and/or a

statement indicating approval of this

research

•

Indicate the form of consent obtained

(written/oral) or the reason that consent was

not obtained (e.g. the data were analyzed

anonymously)

•

Animal Research (involving vertebrate

animals, embryos or tissues)

Provide the name of the Institutional Animal

Care and Use Committee (IACUC) or other

relevant ethics board that reviewed the

study protocol, and indicate whether they

approved this research or granted a formal

waiver of ethical approval

•

Include an approval number if one was

obtained

•

If the study involved non-human primates,

add additional details about animal welfare

and steps taken to ameliorate suffering

•

If anesthesia, euthanasia, or any kind of

animal sacrifice is part of the study, include

briefly which substances and/or methods

were applied

•

Field Research

Include the following details if this study

involves the collection of plant, animal, or

other materials from a natural setting:

• Field permit number

Name of the institution or relevant body that

granted permission

•

Data Availability

Authors are required to make all data

underlying the findings described fully

available, without restriction, and from the

time of publication. PLOS allows rare

exceptions to address legal and ethical

concerns. See the PLOS Data Policy and

FAQ for detailed information.

Yes - all data are fully available without restriction

*Powered by Editorial Manager® and ProduXion Manager® from Aries Systems Corporation*

A Data Availability Statement describing

where the data can be found is required at

submission. Your answers to this question

constitute the Data Availability Statement

and will be published in the article, if

accepted.

Important: Stating ‘data available on request

from the author’ is not sufficient. If your data

are only available upon request, select ‘No’ for

the first question and explain your exceptional

situation in the text box.

Do the authors confirm that all data

underlying the findings described in their

manuscript are fully available without

restriction?

Describe where the data may be found in

full sentences. If you are copying our

sample text, replace any instances of XXX

with the appropriate details.

If the data are held or will be held in a

public repository, include URLs,

accession numbers or DOIs. If this

information will only be available after

acceptance, indicate this by ticking the

box below. For example: All XXX files

are available from the XXX database

(accession number(s) XXX, XXX.).

•

If the data are all contained within the

manuscript and/or Supporting

Information files, enter the following:

All relevant data are within the

manuscript and its Supporting

Information files.

•

If neither of these applies but you are

able to provide details of access

elsewhere, with or without limitations,

please do so. For example:

Data cannot be shared publicly because

of [XXX]. Data are available from the

XXX Institutional Data Access / Ethics

Committee (contact via XXX) for

researchers who meet the criteria for

access to confidential data.

The data underlying the results

presented in the study are available

from (include the name of the third party

•

All relevant data are within the manuscript and its Supporting Information files.

*Powered by Editorial Manager® and ProduXion Manager® from Aries Systems Corporation*

and contact information or URL).

This text is appropriate if the data are

owned by a third party and authors do

not have permission to share the data.

•

* typeset

Additional data availability information:

*Powered by Editorial Manager® and ProduXion Manager® from Aries Systems Corporation*

1

**Autonomy of pregnant women in choosing the mode of delivery: A**

**retrospective study**

**Autonomy in choosing the mode of delivery**

Carlos Henrique Mascarenhas Silva1, Cláudia Lourdes Soares Laranjeira2, Carolina

Soares Barros de Melo3, Lorena Ventura Brandão4, Gabriela Costa Oliveira5, Augusto

Henrique Fulgêncio Brandão6, Rui Nunes7

1 - PhD student in Bioethics, Faculty of Medicine of the University of Porto, Portugal;

Head of Obstetrics and Gynecology Unit - Mater Dei Health Network, Brazil

2 - Head of Obstetrics and Gynecology Unit - Mater Dei Health Network, Brazil;

Assistant Professor, Department of Women's Health, School of Medical Sciences of

Minas Gerais, Belo Horizonte, PhD student in Federal University of Minas Gerais, Brazil

3 - Obstetrics and Gynecology Unit - Mater Dei Health Network, Brazil

4 - Obstetrics and Gynecology Unit - Mater Dei Health Network, Brazil

5 - Resident in Obstetrics and Gynecology Unit - Mater Dei Health Network, Brazil

6 - Adjunct Professor - Obstetrics and Gynecology Department - Federal University of

Minas Gerais; Obstetrics and Gynecology Unit - Mater Dei Health Network, Brazil

7 - Full Professor, Faculty of Medicine of the University of Porto, Portugal

*Corresponding author

Email: carloshenrique@materdei.com.br

These authors also contributed equally to this work

Manuscript Click here to access/download;Manuscript;autonomy plos

one.docx

2

**Abstract:**

Obstetrics is undergoing transformation and committing to ensuring the autonomy of

pregnant women in decisions related to birth based on scientific information. Vaginal

delivery results from the physiological process of birth, but medicine has evolved to

include obstetric surgeries that are safe and have few perioperative complications,

especially when cesarean section is performed from 39 weeks of gestational age. Thus,

the question is whether the clinician should continue to interfere with the freedom of the

pregnant woman to choose the mode of delivery when trying to persuade her to choose

vaginal delivery for the birth of her child. To analyze the perinatal complications of the

maternal–fetal binomial of primiparous women subjected to vaginal delivery versus

elective cesarean section. In total, 2,507 deliveries, including 1,807 (72.1%) vaginal

deliveries and 700 (27.9%) cesarean deliveries, were analyzed between 2017 and 2020.

There was no difference in maternal readmission, death, admission to the intensive care

unit, Apgar score <7 in the 5th minute of life, maternal blood transfusion or comorbidities

of mothers or newborns between the types of delivery. The elective cesarean section

showed less need for therapeutic uterotonics. It was observed that elective cesarean

section in primigravidae does not present a higher risk of complications than vaginal

delivery. This guarantees, therefore, the autonomy and right of the individual to choose

the mode of delivery.

**Introduction:**

Although pregnancy and parturition are natural processes, negative or positive

experiences during the postpartum pregnancy cycle can influence current or future

3

pregnancy, triggering profound transformations.1 Current studies based on scientific

evidence have shown that the presence of the family in the environment and the active

involvement of the patient in decision-making and behaviors are able to improve the

clinical outcomes of the pregnant woman and newborn, positively enriching the

experience of pregnancy.1-2

Autonomy is one of the four pillars of bioethics and is fundamental for humanized

and respectful care at birth.3 Obstetric care is undergoing a transformation process in an

attempt to ensure autonomy and places the parturient as the center of the entire process.4

Shared responsibility is possible through dialog and trust with the care team from

the beginning of prenatal care until the time of delivery and depends on cultural, social,

emotional and economic conditions, among others. For this, the woman needs to be

properly informed and welcomed and to understand what choices should be made based

on quality technical scientific knowledge together with the care team.5

Vaginal delivery is often neglected by the woman due to fear of pain, fear of not

being able to give birth, insecurity about the future sexual life and possible perineal

complications. In addition, for vaginal delivery, she must be empowered and emotionally

and physically prepared for the event, even though it is physiological.6.7

Insecurities and doubts should be addressed during obstetric care; however, even

after knowing all the risks and benefits, based on quality information, the patient may

remain determined to have an abdominal delivery. In this case, the choice of cesarean

section should be respected, without prejudice or attempts at coercion.8

Medical-hospital care in Brazil is struggling to achieve the statistical data

recommended by the World Health Organization (WHO), which claimed in 1985 that the

mode of vaginal delivery was the most beneficial and advised that the percentage of

cesarean sections should not exceed 15% of the total number of births.9 Thereafter,

vaginal delivery has been imposed in maternity wards, even though medicine has

advanced rapidly to the point of achieving safe surgeries with low rates of perinatal

complications, especially for elective cesarean sections of pregnant women at the usual

risk.10 This leads to questions regarding the extent to which the autonomy of the pregnant

woman is respected in the choice of delivery route.

Currently, there are several protocols discussing the issue of on-demand cesarean

section, and they advise its implementation starting at 39 weeks of gestation to reduce the

rates of neonatal respiratory distress and improve morbidity and mortality, thereby

making elective abdominal delivery safe in the medium and short term to the level of

vaginal delivery.11-13.

According to resolution number 2.144 in 2016 and the reaffirmation with new

additions to resolution 2.284 in 2020, the Federal Council of Medicine has advised that

the doctor adhere to the patient's desire to perform the cesarean section as long as the

pregnancy has completed a minimum of 39 weeks of gestation (273 days) and the decision

is documented in medical records along with a signed informed consent form. This

guarantees the woman’s autonomy and adheres to the principle of nonmaleficence by

ensuring fetal maturity to reduce neonatal complications.14.15

There are also national laws and projects such as PL 3.635/2019 that defend the

right to elective cesarean section, respecting the autonomy of the patient and the care

teams in both public and private services in the country.16

We also have an important defining basis for the autonomy of patients in the

choice of the mode of delivery that was discussed in the United Nations International

Convention on the Civil and Political Rights of Women, in the chapter on respect for the

reproductive rights of women and in the scope of the nondiscrimination policy, associated

with article 1 of the Convention for the Elimination of Forms of Discrimination against

Women (CEDAW), in 1979. This convention stipulates that “any distinction, exclusion

or restriction on the basis of sex which has the effect or purpose of impairing or nullifying

the recognition, enjoyment or exercise by women, irrespective of her marital status, on

the basis of equality between men and women, of human rights or fundamental freedoms

in the political, economic, social, cultural civil or any other field” should be avoided.

Thus, ensuring the right of women to choose their mode of delivery is in absolute

agreement with the proposals contained in the statements cited, since capable individuals

have the right to consider their options and make their own choices, whether consenting

to or refusing medical treatment.17

Thus, the objective of the present study is to compare the perinatal complications

of the mother and newborn among primiparous women subjected to term vaginal delivery

or elective cesarean section after 39 weeks of gestational age, ensuring respect for

autonomy in the choice of delivery route, in a private quaternary hospital in Belo

Horizonte.

**Methodology**

This was a retrospective observational study. Data were collected after approval

by the ethics committee: CAAE 36135120.5.0000.5128, opinion 4,400,669.

A total of 2,507 medical records of women admitted to the care of a team of

gynecologists and obstetricians who followed institutional conduct discussed in periodic

clinical meetings with critical analysis in a private hospital with a quaternary health care

level between January 2017 and March 2020 were analyzed, with 1,807 (72.1%) having

vaginal deliveries and 700 (27.9%) having elective cesarean sections.

The inclusion criteria were as follows: presence of a freely signed informed

consent form specific for obstetric care, according to the choice made at the time of

hospitalization; primigravida status; gestational age above 37 weeks for vaginal birth or 39

weeks for elective cesarean section; fetus without malformations; and cephalic

presentation.

The outcomes analyzed included gestational age, type of anesthesia, newborn

weight, Apgar score less than seven in the fifth minute, referral to room-in or admission to

a neonatal or adult intensive care unit (ICU) in the first 24 hours of life, immediate

breastfeeding, maternal death and/or early neonatal death, maternal and/or neonatal

readmission, increased uterine bleeding, need for blood transfusion, comorbidities, use of

uterotonic drugs and maternal hospitalization time.

Categorical variables are presented as absolute and relative frequencies, and

numerical variables are presented as the mean ± standard deviation and median (1st

quartile-3rd quartile). The numerical variables were subjected to the Anderson–Darling

normality test, and Student’s t-test and the Mann–Whitney test were used to compare

means between groups. The associations between categorical variables were evaluated by

the chi-square test (with a simulated p value in the case of expected frequencies less than

1).

A significance level of 1% was considered, and the analyses were performed

using R version 4.0.3 software.

**Results**

A total of 2,507 births were analyzed, of which 1,807 (72.1%) were vaginal births

and 700 (27.9%) were cesarean sections.

Table 1 shows the comparison of birth characteristics.

7

Table 1 - Comparison of characteristics and outcomes of vaginal births and cesarean

sections

**General**

(n=2.507)

**Vaginal delivery**

(n=1.807)

**Cesarean section**

(n=700)

**P value**

**Maternal age**

(n=1.515)

30.6 ± 5.0

31.0 (28.0–34.0)

30.0 ± 4.9

30.0 (27.0–34.0)

32.1 ± 4.8

32.0 (29.0–35.0)

<0.001M

**Accommodation**

(n=1.244)

- -

Set 1.244 (100.0) 692 (100.0) 552 (100.0)

ICU 0 (0.0) 0 (0.0) 0 (0.0)

**Anesthesia**

(n=2.253)

<0.001Q

No 12 (0.5) 12 (0.7) 0 (0.0)

Location 3 (0.1) 3 (0.2) 0 (0.0)

Epidural 1.779 (79.0) 1.777 (98.3) 2 (0.4)

Spinal anesthesia 459 (20.4) 15 (0.8) 444 (99.6)

**Gestational age**

(n=2.498)

39.4 ± 1.0

39.5 (38.7–40.1)

39.3 ± 1.0

39.3 (38.6–40.0)

39.7 ± 0.8

40.0 (39.1–40.1)

<0.001M

**Weight** (n=2.485)

3.213.1 ± 382.5

3.210.0 (2.960.0–

3.460.0)

3.155.8 ± 365.3

3.160.0 (2.920.0–

3.405.0)

3.360.3 ± 386.8

3.350.0 (3.100.0–

3.620.0)

<0.001T

**PICU** 0.043Q

Yes 72 (2.9) 60 (3.3) 12 (1.7)

No 2.435 (97.1) 1.747 (96.7) 688 (98.3)

**5-minute APGAR** 0.063Q

< 7 18 (0.7) 17 (0.9) 1 (0.1)

≥ 7 2.489 (99.3) 1.790 (99.1) 699 (99.9)

8

**Immediate**

**Breast feeding**

<0.001Q

Yes 2.414 (96.3) 1.714 (94.9) 700 (100.0)

No 93 (3.7) 93 (5.1) 0 (0.0)

**Maternal death** - -

Yes 0 (0.0) 0 (0.0) 0 (0.0)

No 2.507 (100.0) 1.807 (100.0) 700 (100.0)

**Neonatal death** - -

Yes 0 (0.0) 0 (0.0) 0 (0.0)

No 2.504 (100.0) 1.804 (100.0) 700 (100.0)

**Maternal**

**readmission**

0.569Q

Yes 32 (1.3) 25 (1.4) 7 (1.0)

No 2.475 (98.7) 1.782 (98.6) 693 (99.0)

**Neonatal**

**readmission**

(n=2.499)

<0.001Q

Yes 183 (7.3) 177 (9.8) 6 (0.9)

No 2.316 (92.7) 1.622 (90.2) 694 (99.1)

**Uterine bleeding** <0.001Q

Usual 2.222 (88.6) 1.574 (87.1) 648 (92.6)

Increased 285 (11.4) 233 (12.9) 52 (7.4)

**Transfusion** >0.999Q

Yes 7 (0.3) 5 (0.3) 2 (0.3)

No 2.500 (99.7) 1.802 (99.7) 698 (99.7)

**Use of uterotonic**

(n=2.506)

<0.001Q

9

Prophylactic 1.660 (66.2) 1.014 (56.1) 646 (92.3)

Therapeutic 845 (33.7) 792 (43.9) 53 (7.6)

Puerperal

hysterectomy

1 (0.0) 0 (0.0) 1 (0.1)

**Comorbidities**

(n=2.496)

0.146Q

Yes 564 (22.6) 394 (21.8) 170 (24.6)

No 1.932 (77.4) 1.412 (78.2) 520 (75.4)

M Mann–Whitney test; T Student’s t-test; Q Chi-square test

The maternal age of the analyzed patients ranged from 27 to 35 years; no

puerperal woman was referred to the ICU, and maternal or neonatal death was not

documented in the analyzed sample.

There was no significant difference in admission to a pediatric intensive care unit

(PICU), Apgar score lower than seven in the fifth minute of life, maternal readmission,

need for blood transfusion or percentage of comorbidities between groups.

There was a difference in the mean gestational age between the vaginal delivery

group (38 weeks + 6 days to 40 weeks) and the cesarean section group (39 weeks + 7 days

to 40 weeks + 1 day).

The vaginal delivery group had more neonatal readmission and higher rates of

puerperal bleeding and consequently a greater need for therapeutic use of uterotonics.

Only one patient, representative of the elective cesarean section, underwent puerperal

hysterectomy due to failure of the clinical treatment for puerperal hemorrhage.

The elective cesarean section group had a higher rate of immediate breastfeeding

than the vaginal delivery group.

10

The mean length of hospital stay after vaginal delivery was 24 to 48 hours, and

after elective cesarean section, it was 48 to 72 hours.

Table 2 shows other information on vaginal births.

Table 2 - Other characteristics of vaginal births

**Statistics**

**Perineal lesion** (n=1.790)

1st grade 264 (14.7)

2nd grade 627 (35.0)

3rd grade 42 (2.3)

4th grade 5 (0.3)

Neck 4 (0.2)

No 848 (47.4)

**Episiotomy** (n=1.806)

Yes 1.376 (76.2)

No 430 (23.8)

**Hospital stay (hours)** – (n=1.805)

41.6 ± 17.6

48.0 (24.0–48.0)

Table 3 shows the distribution of the Robson classification in all pregnant women

and by type of delivery.

Table 3 - Distribution of the Robson classification by type of delivery

**General Vaginal delivery Cesarean section**

11

(n=2.506) (n=1.807) (n=699)

**Robson Classification**

Group 1 1.514 (60.4) 1.369 (75.8) 145 (20.7)

Group 2 989 (39.5) 439 (24.2) 553 (78.8)

Group 3 0 (0.0) 0 (0.0) 0 (0.0)

Group 4 0 (0.0) 0 (0.0) 0 (0.0)

**Discussion:**

The differentiating feature of the study is the selection of elective cesarean

sections in nulliparous women after 39 weeks of gestational age. Most articles that show

worse outcomes in the maternal–fetal binomial did not do this. Instead, their results are

based on the total number of cesarean sections without exclusions of intrapartum

emergency cesarean section, iterativity, maternal or fetal comorbidity-related indications

for the procedure or gestational age, which can increase maternal and neonatal morbidity

and mortality compared to term spontaneous vaginal delivery.

The present study does not aim to encourage or promote the practice of cesarean

sections in an indiscriminate manner, maintaining the recommendation of the American

College of Gynecology and Obstetrics (ACOG) to encourage, but not obligate, the

pregnant woman to undergo vaginal delivery but rather to address future aspects such as

the risk of subsequent surgeries for those who choose to have more children, placenta

previa, placental accreta and other long-term obstetric risks.12

The results allow only for the corroboration of respect for the autonomy of the

pregnant woman, without judgment or criticism from the team, guaranteeing her right of

choice due to the absence of greater maternal or fetal perioperative risks in the short and

12

medium term, which are still erroneously used as an argument in environments that

persuade the pregnant woman to undergo vaginal delivery, without ensuring her autonomy

during parturition.

Another notable point of the study is that ensuring the autonomy of the pregnant

woman over the delivery route did not negatively impact the rates of cesarean section,

since the total percentage of cesarean sections was only 27.5% of all births, even in a

supplementary system of health that respects the autonomy of women.

Although the vaginal delivery group had more neonatal readmissions, the main

reason was late neonatal jaundice. Perinatal jaundice is associated with several factors,

such as the presence of gestational diabetes mellitus, preeclampsia, umbilical cord

clamping time, late elimination of meconium, blood incompatibility and significant

weight loss during the first week, and is not directly related to the type of delivery.18.19

The main comorbidities in decreasing order presented in the vaginal delivery

group were hypothyroidism, preeclampsia without severity criteria, controlled chronic

hypertension (CCH), gestational diabetes mellitus controlled with diet and asthma. In the

cesarean section, the symptoms were hypothyroidism, asthma, controlled dietary

gestational diabetes mellitus and gestational hypertension (GH).

The vaginal delivery group had a higher incidence of puerperal bleeding, although

it is not possible to conclude that cesarean section is preventive because it was not

considered the highest rate of preeclampsia and CCH in the vaginal delivery group, which

are known risk factors for puerperal bleeding. Moreover, the labor time and the incidence

of induction in the vaginal delivery group were not analyzed because prolonged births are

associated with uterine hypotonia, the main cause of increased bleeding.20

The elective cesarean section group had a higher rate of breastfeeding in the first

13

hour of life, which may be indirectly related to the higher incidence of puerperal

hemorrhage in the vaginal delivery group because breastfeeding is not stimulated during

the protocol and use of uterotonics. This isolated finding agrees with the data found in the

literature, which show a lower rate of early breastfeeding because the cesarean section

performed before the onset of labor interferes with the release of oxytocin and,

consequently, lactogenesis, making it difficult to initiate breastfeeding.21

Although elective cesarean section hinders early lactation, evidence suggests that

it has no effect on the interruption of breastfeeding before 6 months of life and does not

impact the occurrence of respiratory infections, atopy or overweight/obesity in the first

year of life.22

Liu X. et al. conducted a retrospective cohort in 2015 with an analysis of 66,226

nulliparous women between 2007 and 2013 who had spontaneous vaginal delivery

comparing the outcomes with the group subjected to elective cesarean section. There was

no significant difference in admission to the ICU, puerperal hemorrhage, maternal

infection, thromboembolism, organ injury or perinatal mortality. The neonates subjected

to vaginal delivery had a higher rate of birth injuries, neonatal infection, hypoxic ischemic

encephalopathy, and meconium aspiration. Conversely, the elective cesarean section

showed a higher rate of respiratory distress syndrome, though the minimum gestational

age of 39 weeks for abdominal delivery was not observed. The study corroborates the data

found in the present study, allowing us to argue the safety of the elective procedure

compared to vaginal delivery.23

Among the limiting factors of the study is the sample size and its heterogeneity,

although the analyses were performed after adjustment, considering the proportions in

each group. Another bias is that the data are from only one medical center with all the

available structures and trained staff; therefore, more multicenter studies with different

14

realities are needed to strengthen the scientific evidence.

However, although it is not possible to state that elective cesarean section is

protective, it can be concluded that it does not worsen the maternal or fetal outcome in the

short or medium term. It is concluded that elective abdominal delivery in a controlled

environment is as safe as vaginal delivery and, therefore, should be respected when this is

the woman's choice. It is also important that the obstetrician is the guarantor of the choices

and desires of the pregnant women in the context of maternal health care.

**Conclusion**

In the full exercise of her reproductive rights, the woman has the right to participate

in the decision-making process regarding the childbirth method, after receiving appropriate

guidance. This must be treated as a basic right in the exercise of her citizenship.

Thus, it is essential that the information is transmitted continuously to her throughout her

pregnancy, at prenatal consultations, so that the choice is also based on adequate

knowledge.

The study also shows quite clearly that the risks and complications related to caesarean

delivery are similar to those of vaginal delivery, increasing the ethical obligation of

the obstetrician to offer both possibilities to the pregnant woman. That is, it is possible to

include the woman in the decision-making process regarding her delivery, and such

choices do not affect the safety of birth.

It is important to note that the clinical care practice of offering and making available

the choice of delivery route for women at the birth of their children should also have,

as a practical and conceptual basis, the bioethical precept of *justice*, and the choice should

be offered equally to all pregnant women, including users of the public (Sistema Único

15

de Saúde-SUS) or private (Health Plan Operators) systems, regardless of cultural, religious

or socioeconomic factors.

Future studies may add to the evaluation of women regarding respect for their

autonomy in making this choice during the prenatal period and during their hospitalization

at maternity hospitals for their deliveries, in addition to evaluating their satisfaction

with this choice, using the aid of research already that has already validated assessing this

satisfaction with the care provided by the care team in maternity wards and hospitals.

**References**

1- Shakibazadeh E, Namadian M, Bhoren M, Vogel JP, et al. Respectful care during

childbirth in health facilities globally: a qualitative evidence synthesis. BJOG. 2018;

125(8) 932-942.

2- Vogel JP, Bhoren MA, Tunçalp Ö OT, Gülmezoglu AM. Promoting respect and

preventing mistreatment during childbirth. BJOG. 2016; 123 (5):671-674.

3- Yusrita Z. Evaluating the concept of choice in healthcare. Malays J Med Sci. 2017;

24(6):92-6.

4- Benyamini Y, Molcho ML, Dan U, Preis H. Women’s attitudes toward the

medicalization of childbirth and their associations with planned and actual modes os

birth. Women Birth. 2019; 30(5):424-30.

5- Sens MM, Stamm AMNF. Physycians perception of obstetric or institutional violence

in the sobtle dimension of the human and physician-patient relationship. Interface

(Botucatu). 2019; 23:e180487.

Available from:

https://www.scielo.br/j/icse/a/jQy8NkBxxx5Zgw3wrpnPY9n/?lang=en

16

6- Bergholt T, Ostberg B, Legarth J, Weber T. Danish obstetricians’personal preference

and general attitude to elective cesarean section on maternal request: a nation-wide

postal survey. Acta Obstet Gynecol Scand. 2004; (3):83, 262-6.

7- Loke, AY, Davies L, Mak YW. Is it decision of women to choose a cesarean section

as the mode of birth? A review of literature on the views of stakeholders. Pregnancy

and Childbirth. 2019; (286):19, 1-9.

8- Caughey AB, Cahill AG, Guise J, Rouse DJ. Safe prevention of the primary cesarean

birth. Am L Obstetrics Gynecol. 2014; (3):210, 179-93.

9- World Health Organisation (WHO). Appropriate Technology for birth. The Lancet.

1985; 326(8452):436-7.

10- Yang XJ, Sun SS. Comparison of maternal and fetal complication in elective and

emergency cesarean section: a systematic review and meta-analysis. Arch Gynecol

Obstet. 2017; 296:503-12.

11- National Institute for Health and Care Excellence (NICE). Caesarean Birth [NG192]:

NICE; 2021. Available from: https://www.nice.org.uk/guidance/ng192

12- The American College of Obstetricians and Gynecologists. Committee on Obstetric

Practice. Cesarean Delivery on maternal request. 2013 (Reaffirmed 2019). No 761.

Obstet Gynecol 2013; 133(1): e73-e77;

13- Prediger B, Mathes T, Polus S, Glastt A, Bühn S et al. Systematic review and time response

meta-analysis on the optimal timing of elective caesarean section for best

maternal and neonatal health outcomes. BMC pregnancy Childbirth. 2020;20(1):395.

14- Conselho Federal de Medicina. Resolução CFM n˚ 2.144/2016; Publicada do D.O.U

de 22 de junho de 2020, seção I, p 138.

15- Conselho Federal de Medicina. Resolução CFM n˚ 2.284/2020; Publicada do D.O.U

de 22 de outubro de 2020, seção I, p 143.

17

16- BRASIL Câmara dos Deputados. Projeto de Lei n˚3.635, de 2019. Altera o PL n˚

4126/2015 que garante à gestante a possibilidade pelo parto cesariano, a partir da

trigésima nona semana de gestação, bem como a analgesia, mesmo quando escolhido

o parto normal. Brasília: Câmara dos Deputados, 2019. Available

from: https://www.camara.leg.br/proposicoesWeb/prop_mostrarintegra?codteor=178

0435. Access in: 07 july, 2021.

17- Larsen, Ann-Claire. Women’s Rights in the Health Care System: Caesarean Delivery

on Maternal Request. Journal of Law and Medicine. 2018; (25) 448-464.

18- Araújo BF. Manejo da icterícia no recém-nascido. In Ferreira JP, organizador.

Pediatria: diagnóstico e tratamento. Porto alegre: Artmed; 2005. P39-45

19- Olusanya BO, Kaplan M, Hansen TWR. Neonatal hyperbilirubinaemia: global

perspective. The Lancet Child & Adolescent Health. 2018;2(8):610-20.

20- Papazian J, Kacmar RM. Obstetric hemorrhage: prevention, recognition, and

Treatment. Adv Anesth. 2017; 35(1):65-93.

21- Hobbs AJ, Mannion CA, McDonald SW, Brockway M, Tough SC. The impact of

caesarean section on breastfeeding initiation, duration, and difficulties in the first four

months postpartum. BMC Pregnancy Childbirth. 2016; 16(1):90

22- Ferrari AP, Machado MCHS, Tonete VLP, Parada CMGL. Effects of elective cesarean

section on outcome in the first year of life: a cohort study. Esc Anna Nery.

2021;25(3):e20200273.

23- Liu X, Landon, MB, Cheng W, Chen Y. Cesarean delivery on maternal request in

china: what are the risks and benefits. Am J Obstet Gynecol. 2015; 212(6):817.e1-9.
