# Supplementary material for: Perinatal complications of the maternal–Fetal dyad in primiparous women subjected to vaginal delivery versus elective cesarean section: A retrospective study of clinical results associated with bioethical precepts
Source: PLoS One. 2023 Oct 17;18(10):e0292846. doi: 10.1371/journal.pone.0292846 (PMC10581477; doi:10.1371/journal.pone.0292846)
Supplement: Attachment — Submitted filename: Response to Reviewers.docx [file pone.0292846.s002.docx]

Belo Horizonte, January 20^th^, 2023

Dear academic editor and reviewer(s):

Viemos através desta carta agradecer aos comentários e as sugestões que auxiliaram na revisão do trabalho e que certamente qualificam e conferem melhorias em nosso texto.

Informamos que as alterações sugeridas pelo academic editor and reviewer(s) foram cumpridas.

A seguir, são feitos alguns esclarecimentos:

Os nomes dos arquivos e a formatação foram corrigidos de acordo com os modelos da plos one.

Foi incluída na metodologia, a informação de dispensa do termo de consentimento livre esclarecido (TCLE) pelo comitê de ética.

Em relação ao financiamento: The author(s) received no specific funding for this work. The funders had no role in study design, data collection and analysis, decision to publish, or preparation of the manuscript, este dado também foi included na revised cover letter.

A primeira referência bibliográfica que havia sido retratada, foi substituída por outra mais atual.

A discussão e a conclusão estão mais claras sobre os dados incluirem apenas primiparous women no estudo, como sugerido pelo Reviewer #1:

Aguardamos a resposta final e estamos dispostos a esclarecer quaisquer dúvidas.

Cordialmente,

Os autores
