# Supplementary material for: Perinatal complications of the maternal–Fetal dyad in primiparous women subjected to vaginal delivery versus elective cesarean section: A retrospective study of clinical results associated with bioethical precepts
Source: PLoS One. 2023 Oct 17;18(10):e0292846. doi: 10.1371/journal.pone.0292846 (PMC10581477; doi:10.1371/journal.pone.0292846)
Supplement: Attachment — Submitted filename: Manuscript (2).docx [file pone.0292846.s003.docx]

**Autonomy of pregnant women in choosing the mode of delivery: A retrospective study**

Carlos Henrique Mascarenhas Silva^1^**^¶^***, Cláudia Lourdes Soares Laranjeira^2 ¶^, Carolina Soares Barros de Melo^3^**^¶^**, Lorena Ventura Brandão^4&^, Gabriela Costa Oliveira^5&^, Augusto Henrique Fulgêncio Brandão^6&^, Rui Nunes^7^**^¶^**

1 - PhD student in Bioethics, Faculty of Medicine of the University of Porto, Portugal;

Head of Obstetrics and Gynecology Unit - Mater Dei Health Network, Brazil

2 - Head of Obstetrics and Gynecology Unit - Mater Dei Health Network, Brazil; Assistant Professor, Department of Women's Health, School of Medical Sciences of Minas Gerais, Belo Horizonte, PhD student in Federal University of Minas Gerais, Brazil

3 - Obstetrics and Gynecology Unit - Mater Dei Health Network, Brazil

4 - Obstetrics and Gynecology Unit - Mater Dei Health Network, Brazil

5 - Resident in Obstetrics and Gynecology Unit - Mater Dei Health Network, Brazil

6 - Adjunct Professor - Obstetrics and Gynecology Department - Federal University of Minas Gerais; Obstetrics and Gynecology Unit - Mater Dei Health Network, Brazil

7 - Full Professor, Faculty of Medicine of the University of Porto, Portugal

*Corresponding author

Email: [carloshenrique@materdei.com.br](mailto:carloshenrique@materdei.com.br)

**^¶^** These authors also contributed equally to this work

& These co-authors also contributed equally to this work.

**Abstract:**

Obstetrics is undergoing transformation and committing to ensuring the autonomy of pregnant women in decisions related to birth based on scientific information. Vaginal delivery results from the physiological process of birth, but medicine has evolved to include obstetric surgeries that are safe and have few perioperative complications, especially when cesarean section is performed from 39 weeks of gestational age. Thus, the question is whether the clinician should continue to interfere with the freedom of the pregnant woman to choose the mode of delivery when trying to persuade her to choose vaginal delivery for the birth of her child. To analyze the perinatal complications of the maternal–fetal binomial of primiparous women subjected to vaginal delivery versus elective cesarean section. In total, 2,507 deliveries, including 1,807 (72.1%) vaginal deliveries and 700 (27.9%) cesarean deliveries, were analyzed between 2017 and 2020.

There was no difference in maternal readmission, death, admission to the intensive care unit, Apgar score <7 in the 5th minute of life, maternal blood transfusion or comorbidities of mothers or newborns between the types of delivery. The elective cesarean section showed less need for therapeutic uterotonics. It was observed that elective cesarean section in primigravidae does not present a higher risk of complications than vaginal delivery. This guarantees, therefore, the autonomy and right of the individual to choose the mode of delivery.

**Introduction**

Although pregnancy and parturition are natural processes, negative or positive experiences during the postpartum pregnancy cycle can influence current or future pregnancy, triggering profound transformations.^1^ Current studies based on scientific evidence have shown that the presence of the family in the environment and the active involvement of the patient in decision-making and behaviors are able to improve the clinical outcomes of the pregnant woman and newborn, positively enriching the experience of pregnancy.^1-2^

Autonomy is one of the four pillars of bioethics and is fundamental for humanized and respectful care at birth.^3^ Obstetric care is undergoing a transformation process in an attempt to ensure autonomy and places the parturient as the center of the entire process.^4^

Shared responsibility is possible through dialog and trust with the care team from the beginning of prenatal care until the time of delivery and depends on cultural, social, emotional and economic conditions, among others. For this, the woman needs to be properly informed and welcomed and to understand what choices should be made based on quality technical scientific knowledge together with the care team.^5^

Vaginal delivery is often neglected by the woman due to fear of pain, fear of not being able to give birth, insecurity about the future sexual life and possible perineal complications. In addition, for vaginal delivery, she must be empowered and emotionally and physically prepared for the event, even though it is physiological.^6.7^

Insecurities and doubts should be addressed during obstetric care; however, even after knowing all the risks and benefits, based on quality information, the patient may remain determined to have an abdominal delivery. In this case, the choice of cesarean section should be respected, without prejudice or attempts at coercion.^8^

Medical-hospital care in Brazil is struggling to achieve the statistical data recommended by the World Health Organization (WHO), which claimed in 1985 that the mode of vaginal delivery was the most beneficial and advised that the percentage of cesarean sections should not exceed 15% of the total number of births.^9^ Thereafter, vaginal delivery has been imposed in maternity wards, even though medicine has advanced rapidly to the point of achieving safe surgeries with low rates of perinatal complications, especially for elective cesarean sections of pregnant women at the usual risk.^10^ This leads to questions regarding the extent to which the autonomy of the pregnant woman is respected in the choice of delivery route.

Currently, there are several protocols discussing the issue of on-demand cesarean section, and they advise its implementation starting at 39 weeks of gestation to reduce the rates of neonatal respiratory distress and improve morbidity and mortality, thereby making elective abdominal delivery safe in the medium and short term to the level of vaginal delivery.^11-13.^

According to resolution number 2.144 in 2016 and the reaffirmation with new additions to resolution 2.284 in 2020, the Federal Council of Medicine has advised that the doctor adhere to the patient's desire to perform the cesarean section as long as the pregnancy has completed a minimum of 39 weeks of gestation (273 days) and the decision is documented in medical records along with a signed informed consent form. This guarantees the woman’s autonomy and adheres to the principle of nonmaleficence by ensuring fetal maturity to reduce neonatal complications.^14.15^

There are also national laws and projects such as PL 3.635/2019 that defend the right to elective cesarean section, respecting the autonomy of the patient and the care teams in both public and private services in the country.^16^

We also have an important defining basis for the autonomy of patients in the choice of the mode of delivery that was discussed in the United Nations International Convention on the Civil and Political Rights of Women, in the chapter on respect for the reproductive rights of women and in the scope of the nondiscrimination policy, associated with article 1 of the Convention for the Elimination of Forms of Discrimination against Women (CEDAW), in 1979. This convention stipulates that “any distinction, exclusion or restriction on the basis of sex which has the effect or purpose of impairing or nullifying the recognition, enjoyment or exercise by women, irrespective of her marital status, on the basis of equality between men and women, of human rights or fundamental freedoms in the political, economic, social, cultural civil or any other field” should be avoided. Thus, ensuring the right of women to choose their mode of delivery is in absolute agreement with the proposals contained in the statements cited, since capable individuals have the right to consider their options and make their own choices, whether consenting to or refusing medical treatment.^17^

Thus, the objective of this study is to compare the perinatal complications of the mother and newborn among primiparous women subjected to term vaginal delivery or elective cesarean section after 39 weeks of gestational age, ensuring respect for autonomy in the choice of delivery route, in a private quaternary hospital in Belo Horizonte.

**Methodology**

This was a retrospective observational study. Data were collected after approval by the ethics committee: CAAE 36135120.5.0000.5128, opinion 4,400,669.

A total of 2,507 medical records of women admitted to the care of a team of gynecologists and obstetricians who followed institutional conduct discussed in periodic clinical meetings with critical analysis in a private hospital with a quaternary health care level between January 2017 and March 2020 were analyzed, with 1,807 (72.1%) having vaginal deliveries and 700 (27.9%) having elective cesarean sections.

The inclusion criteria were as follows: presence of a freely signed informed consent form specific for obstetric care, according to the choice made at the time of hospitalization; primigravida status; gestational age above 37 weeks for vaginal birth or 39 weeks for elective cesarean section; fetus without malformations; and cephalic presentation.

The outcomes analyzed included gestational age, type of anesthesia, newborn weight, Apgar score less than seven in the fifth minute, referral to room-in or admission to a neonatal or adult intensive care unit (ICU) in the first 24 hours of life, immediate breastfeeding, maternal death and/or early neonatal death, maternal and/or neonatal readmission, increased uterine bleeding, need for blood transfusion, comorbidities, use of uterotonic drugs and maternal hospitalization time.

Categorical variables are presented as absolute and relative frequencies, and numerical variables are presented as the mean ± standard deviation and median (1st quartile-3rd quartile). The numerical variables were subjected to the Anderson–Darling normality test, and Student’s t-test and the Mann–Whitney test were used to compare means between groups. The associations between categorical variables were evaluated by the chi-square test (with a simulated p value in the case of expected frequencies less than 1).

A significance level of 1% was considered, and the analyses were performed using R version 4.0.3 software.

**Results**

A total of 2,507 births were analyzed, of which 1,807 (72.1%) were vaginal births and 700 (27.9%) were cesarean sections.

Table 1: Comparison of characteristics and outcomes of vaginal births and cesarean sections

| **Characteristic** | **General**  (n=2,507) | **Vaginal delivery**  (n=1,807) | **Cesarean section**  (n=700) | **P value** |
| --- | --- | --- | --- | --- |
| **Maternal age** (n=1.515) | 30.6 ± 5.0  31.0 (28.0–34.0) | 30.0 ± 4.9  30.0 (27.0–34.0) | 32.1 ± 4.8  32.0 (29.0–35.0) | <0.001^M^ |
| **Accommodation** (n=1.244) |  |  |  | - - |
| Set | 1.244 (100.0) | 692 (100.0) | 552 (100.0) |  |
| ICU | 0 (0.0) | 0 (0.0) | 0 (0.0) |  |
| **Anesthesia** (n=2.253) |  |  |  | <0.001^Q^ |
| No | 12 (0.5) | 12 (0.7) | 0 (0.0) |  |
| Location | 3 (0.1) | 3 (0.2) | 0 (0.0) |  |
| Epidural | 1.779 (79.0) | 1.777 (98.3) | 2 (0.4) |  |
| Spinal anesthesia | 459 (20.4) | 15 (0.8) | 444 (99.6) |  |
| **Gestational age** (n=2.498) | 39.4 ± 1.0  39.5 (38.7–40.1) | 39.3 ± 1.0  39.3 (38.6–40.0) | 39.7 ± 0.8  40.0 (39.1–40.1) | <0.001^M^ |
| **Weight** (n=2.485) | 3.213.1 ± 382.5  3.210.0 (2.960.0–3.460.0) | 3.155.8 ± 365.3  3.160.0 (2.920.0–3.405.0) | 3.360.3 ± 386.8  3.350.0 (3.100.0–3.620.0) | <0.001^T^ |
| **PICU** |  |  |  | 0.043^Q^ |
| Yes | 72 (2.9) | 60 (3.3) | 12 (1.7) |  |
| No | 2.435 (97.1) | 1.747 (96.7) | 688 (98.3) |  |
| **5-minute APGAR** |  |  |  | 0.063^Q^ |
| < 7 | 18 (0.7) | 17 (0.9) | 1 (0.1) |  |
| ≥ 7 | 2.489 (99.3) | 1.790 (99.1) | 699 (99.9) |  |
| **Immediate breastfeeding** |  |  |  | <0.001^Q^ |
| Yes | 2.414 (96.3) | 1.714 (94.9) | 700 (100.0) |  |
| No | 93 (3.7) | 93 (5.1) | 0 (0.0) |  |
| **Maternal death** |  |  |  | - - |
| Yes | 0 (0.0) | 0 (0.0) | 0 (0.0) |  |
| No | 2.507 (100.0) | 1.807 (100.0) | 700 (100.0) |  |
| **Neonatal death** |  |  |  | - - |
| Yes | 0 (0.0) | 0 (0.0) | 0 (0.0) |  |
| No | 2.504 (100.0) | 1.804 (100.0) | 700 (100.0) |  |
| **Maternal readmission** |  |  |  | 0.569^Q^ |
| Yes | 32 (1.3) | 25 (1.4) | 7 (1.0) |  |
| No | 2.475 (98.7) | 1.782 (98.6) | 693 (99.0) |  |
| **Neonatal readmission**  (n=2.499) |  |  |  | <0.001^Q^ |
| Yes | 183 (7.3) | 177 (9.8) | 6 (0.9) |  |
| No | 2.316 (92.7) | 1.622 (90.2) | 694 (99.1) |  |
| **Uterine bleeding** |  |  |  | <0.001^Q^ |
| Usual | 2.222 (88.6) | 1.574 (87.1) | 648 (92.6) |  |
| Increased | 285 (11.4) | 233 (12.9) | 52 (7.4) |  |
| **Transfusion** |  |  |  | >0.999^Q^ |
| Yes | 7 (0.3) | 5 (0.3) | 2 (0.3) |  |
| No | 2.500 (99.7) | 1.802 (99.7) | 698 (99.7) |  |
| **Use of uterotonic** (n=2.506) |  |  |  | <0.001^Q^ |
| Prophylactic | 1.660 (66.2) | 1.014 (56.1) | 646 (92.3) |  |
| Therapeutic | 845 (33.7) | 792 (43.9) | 53 (7.6) |  |
| Puerperal hysterectomy | 1 (0.0) | 0 (0.0) | 1 (0.1) |  |
| **Comorbidities** (n=2.496) |  |  |  | 0.146^Q^ |
| Yes | 564 (22.6) | 394 (21.8) | 170 (24.6) |  |
| No | 1.932 (77.4) | 1.412 (78.2) | 520 (75.4) |  |

^M^ Mann–Whitney test; ^T^ Student’s t-test; ^Q^ Chi-square test

The maternal age of the analyzed patients ranged from 27 to 35 years; no puerperal woman was referred to the ICU, and maternal or neonatal death was not documented in the analyzed sample.

There was no significant difference in admission to a pediatric intensive care unit (PICU), Apgar score lower than seven in the fifth minute of life, maternal readmission, need for blood transfusion or percentage of comorbidities between groups.

There was a difference in the mean gestational age between the vaginal delivery group (38 weeks + 6 days to 40 weeks) and the cesarean section group (39 weeks + 7 days to 40 weeks + 1 day).

The vaginal delivery group had more neonatal readmission and higher rates of puerperal bleeding and consequently a greater need for therapeutic use of uterotonics. Only one patient, representative of the elective cesarean section, underwent puerperal hysterectomy due to failure of the clinical treatment for puerperal hemorrhage.

The elective cesarean section group had a higher rate of immediate breastfeeding than the vaginal delivery group.

The mean length of hospital stay after vaginal delivery was 24 to 48 hours, and after elective cesarean section, it was 48 to 72 hours.

Table 2: Other characteristics of vaginal births

| **Outcomes** | **Statistics** |
| --- | --- |
| **Perineal lesion** (n=1.790) |  |
| 1st grade | 264 (14.7) |
| 2nd grade | 627 (35.0) |
| 3rd grade | 42 (2.3) |
| 4th grade | 5 (0.3) |
| Neck | 4 (0.2) |
| No | 848 (47.4) |
| **Episiotomy** (n=1.806) |  |
| Yes | 1.376 (76.2) |
| No | 430 (23.8) |
| **Hospital stay (hours)** – (n=1.805) | 41.6 ± 17.6  48.0 (24.0–48.0) |

Table 3: Distribution of the Robson classification by type of delivery

| **Robson Classification** | **General**  (n=2.506) | **Vaginal delivery**  (n=1.807) | **Cesarean section**  (n=699) |
| --- | --- | --- | --- |
|  |  |  |  |
| Group 1 | 1.514 (60.4) | 1.369 (75.8) | 145 (20.7) |
| Group 2 | 989 (39.5) | 439 (24.2) | 553 (78.8) |
| Group 3 | 0 (0.0) | 0 (0.0) | 0 (0.0) |
| Group 4 | 0 (0.0) | 0 (0.0) | 0 (0.0) |

**Discussion**

The differentiating feature of the study is the selection of elective cesarean sections in nulliparous women after 39 weeks of gestational age. Most articles that show worse outcomes in the maternal–fetal binomial did not do this. Instead, their results are based on the total number of cesarean sections without exclusions of intrapartum emergency cesarean section, iterativity, maternal or fetal comorbidity-related indications for the procedure or gestational age, which can increase maternal and neonatal morbidity and mortality compared to term spontaneous vaginal delivery.

The present study does not aim to encourage or promote the practice of cesarean sections in an indiscriminate manner, maintaining the recommendation of the American College of Gynecology and Obstetrics (ACOG) to encourage, but not obligate, the pregnant woman to undergo vaginal delivery but rather to address future aspects such as the risk of subsequent surgeries for those who choose to have more children, placenta previa, placental accreta and other long-term obstetric risks.^12^

The results allow only for the corroboration of respect for the autonomy of the pregnant woman, without judgment or criticism from the team, guaranteeing her right of choice due to the absence of greater maternal or fetal perioperative risks in the short and medium term, which are still erroneously used as an argument in environments that persuade the pregnant woman to undergo vaginal delivery, without ensuring her autonomy during parturition.

Another notable point of the study is that ensuring the autonomy of the pregnant woman over the delivery route did not negatively impact the rates of cesarean section, since the total percentage of cesarean sections was only 27.5% of all births, even in a supplementary system of health that respects the autonomy of women.

Although the vaginal delivery group had more neonatal readmissions, the main reason was late neonatal jaundice. Perinatal jaundice is associated with several factors, such as the presence of gestational diabetes mellitus, preeclampsia, umbilical cord clamping time, late elimination of meconium, blood incompatibility and significant weight loss during the first week, and is not directly related to the type of delivery.^18.19^

The main comorbidities in decreasing order presented in the vaginal delivery group were hypothyroidism, preeclampsia without severity criteria, controlled chronic hypertension (CCH), gestational diabetes mellitus controlled with diet and asthma. In the cesarean section, the symptoms were hypothyroidism, asthma, controlled dietary gestational diabetes mellitus and gestational hypertension (GH).

The vaginal delivery group had a higher incidence of puerperal bleeding, although it is not possible to conclude that cesarean section is preventive because it was not considered the highest rate of preeclampsia and CCH in the vaginal delivery group, which are known risk factors for puerperal bleeding. Moreover, the labor time and the incidence of induction in the vaginal delivery group were not analyzed because prolonged births are associated with uterine hypotonia, the main cause of increased bleeding.^20^

The elective cesarean section group had a higher rate of breastfeeding in the first hour of life, which may be indirectly related to the higher incidence of puerperal hemorrhage in the vaginal delivery group because breastfeeding is not stimulated during the protocol and use of uterotonics. This isolated finding agrees with the data found in the literature, which show a lower rate of early breastfeeding because the cesarean section performed before the onset of labor interferes with the release of oxytocin and, consequently, lactogenesis, making it difficult to initiate breastfeeding.^21^

Although elective cesarean section hinders early lactation, evidence suggests that it has no effect on the interruption of breastfeeding before 6 months of life and does not impact the occurrence of respiratory infections, atopy or overweight/obesity in the first year of life.^22^

Liu X. et al. conducted a retrospective cohort in 2015 with an analysis of 66,226 nulliparous women between 2007 and 2013 who had spontaneous vaginal delivery comparing the outcomes with the group subjected to elective cesarean section. There was no significant difference in admission to the ICU, puerperal hemorrhage, maternal infection, thromboembolism, organ injury or perinatal mortality. The neonates subjected to vaginal delivery had a higher rate of birth injuries, neonatal infection, hypoxic ischemic encephalopathy, and meconium aspiration. Conversely, the elective cesarean section showed a higher rate of respiratory distress syndrome, though the minimum gestational age of 39 weeks for abdominal delivery was not observed. The study corroborates the data found in the present study, allowing us to argue the safety of the elective procedure compared to vaginal delivery.^23^

Among the limiting factors of the study is the sample size and its heterogeneity, although the analyses were performed after adjustment, considering the proportions in each group. Another bias is that the data are from only one medical center with all the available structures and trained staff; therefore, more multicenter studies with different realities are needed to strengthen the scientific evidence.

However, although it is not possible to state that elective cesarean section is protective, it can be concluded that it does not worsen the maternal or fetal outcome in the short or medium term. It is concluded that elective abdominal delivery in a controlled environment is as safe as vaginal delivery in primigravidae and, therefore, should be respected when this is the woman's choice. It is also important that the obstetrician is the guarantor of the choices and desires of the pregnant women in the context of maternal health care.

**Conclusion**

In the full exercise of her reproductive rights, the woman has the right to participate in the decision-making process regarding the childbirth method, after receiving appropriate guidance. This must be treated as a basic right in the exercise of her citizenship. Thus, it is essential that the information is transmitted continuously to her throughout her pregnancy, at prenatal consultations, so that the choice is also based on adequate knowledge.

The study also shows quite clearly that the risks and complications related to caesarean delivery are similar to those of vaginal delivery in primigravidae, increasing the ethical obligation of the obstetrician to offer both possibilities to the pregnant woman. That is, it is possible to include the woman in the decision-making process regarding her delivery, and such choices do not affect the safety of birth.

It is important to note that the clinical care practice of offering and making available the choice of delivery route for women at the birth of their children should also have, as a practical and conceptual basis, the bioethical precept of *justice*, and the choice should be offered equally to all pregnant women, including users of the public (Sistema Único de Saúde-SUS) or private (Health Plan Operators) systems, regardless of cultural, religious or socioeconomic factors.

Future studies may add to the evaluation of women regarding respect for their autonomy in making this choice during the prenatal period and during their hospitalization at maternity hospitals for their deliveries, in addition to evaluating their satisfaction with this choice, using the aid of research already that has already validated assessing this satisfaction with the care provided by the care team in maternity wards and hospitals.

**References**

1- Nunes AL, Thomaz EBAF, Pinho JRO, Silva L.C. Chagas DC. Childbirth care in Health services affiliated with the stork network in Brazil: the users perspective. Cad Saude Publica; 2022 25;38 (4) PT228921.
2- Vogel JP, Bhoren MA, Tunçalp Ö OT, Gülmezoglu AM. Promoting respect and preventing mistreatment during childbirth. BJOG. 2016; 123 (5):671-674.
3- Yusrita Z. Evaluating the concept of choice in healthcare. Malays J Med Sci. 2017; 24(6):92-6.
4- Benyamini Y, Molcho ML, Dan U, Preis H. Women’s attitudes toward the medicalization of childbirth and their associations with planned and actual modes of birth. Women Birth. 2019; 30(5):424-30.
5- Sens MM, Stamm ANMF. Physicians’ perception of obstetric or institutional violence in the subtle dimension of the human and physician-patient relationship. Interface (Botucatu). 2019; 23:e180487.
6- Bergholt T, Ostberg B, Legarth J, Weber T. Danish obstetricians’ personal preference and general attitude to elective cesarean section on maternal request: a nation-wide postal survey. Acta Obstet Gynecol Scand. 2004; (3):83, 262-6.
7- Loke, AY, Davies L, Mak YW. Is it decision of women to choose a cesarean section as the mode of birth? A review of literature on the views of stakeholders. Pregnancy and Childbirth. 2019; (286):19, 1-9.
8- Caughey AB, Cahill AG, Guise J, Rouse DJ. Safe prevention of the primary cesarean birth. Am L Obstetrics Gynecol. 2014 (Reaffirmed 2019); (3):210, 179-93.
9- World Health Organization (WHO). Appropriate Technology for birth. The Lancet. 1985; 326(8452):436-7.
10- Yang XJ, Sun SS. Comparison of maternal and fetal complication in elective and emergency cesarean section: a systematic review and meta-analysis. Arch Gynecol Obstet. 2017; 296:503-12.
11- National Institute for Health and Care Excellence (NICE). Caesarean Birth [NG192]: NICE; 2021 .Available from: https://www.nice.org.uk/guidance/ng192
12- The American College of Obstetricians and Gynecologists. Committee on Obstetric Practice. Cesarean Delivery on maternal request. 2013 (Reaffirmed 2019). No 761. Obstet Gynecol 2013; 133(1): e73-e77;
13- Prediger B, Mathes T, Polus S, Glastt A, Bühn S et al. Systematic review and time-response meta-analysis on the optimal timing of elective caesarean section for best maternal and neonatal health outcomes. BMC pregnancy Childbirth. 2020;20(1):395.
14- Conselho Federal de Medicina. Resolução CFM n˚ 2.144/2016; Publicada do D.O.U de 22 de junho de 2020, seção I, p 138.
15- Conselho Federal de Medicina. Resolução CFM n˚ 2.284/2020; Publicada do D.O.U de 22 de outubro de 2020, seção I, p 143.
16- BRASIL Câmara dos Deputados. Projeto de Lei n˚3.635, de 2019. Altera o PL n˚ 4126/2015 que garante à gestante a possibilidade pelo parto cesariano, a partir da trigésima nona semana de gestação, bem como a analgesia, mesmo quando escolhido o parto normal. Brasilia: Câmara dos Deputados, 2019. Available from: <https://www.camara.leg.br/proposicoesWeb/prop_mostrarintegra?codteor=1780435>.
17- Larsen, Ann-Claire. Women’s Rights in the Health Care System: Caesarean Delivery on Maternal Request. Journal of Law and Medicine. 2018; 25 (2):448-464.
18- Araújo BF. Manejo da icterícia no recém-nascido. In Ferreira JP, organizador. Pediatria: diagnóstico e tratamento. Porto alegre: Artmed; 2005. P39-45
19- Olusanya BO, Kaplan M, Hansen TWR. Neonatal hyperbilirubinaemia: global perspective. The Lancet Child & Adolescent Health. 2018;2(8):610-20.
20- Papazian J, Kacmar RM. Obstetric hemorrhage: prevention, recognition, and Treatment. Adv Anesth. 2017; 35(1):65-93.
21- Hobbs AJ, Mannion CA, McDonald SW, Brockway M, Tough SC. The impact of caesarean section on breastfeeding initiation, duration, and difficulties in the first four months postpartum. BMC Pregnancy Childbirth. 2016; 16(1):90
22- Ferrari AP, Machado MCHS, Tonete VLP, Parada CMGL. Effects of elective cesarean section on outcome in the first year of life: a cohort study. Esc Anna Nery. 2021; 25(3):e20200273.
23- Liu X, Landon, MB, Cheng W, Chen Y. Cesarean delivery on maternal request in china: what are the risks and benefits. Am J Obstet Gynecol. 2015; 212(6):817.e1-9.
