# Supplementary material for: Perinatal complications of the maternal–Fetal dyad in primiparous women subjected to vaginal delivery versus elective cesarean section: A retrospective study of clinical results associated with bioethical precepts
Source: PLoS One. 2023 Oct 17;18(10):e0292846. doi: 10.1371/journal.pone.0292846 (PMC10581477; doi:10.1371/journal.pone.0292846)
Supplement: Attachment — Submitted filename: Response to Reviewers .doc [file pone.0292846.s004.doc]

Belo Horizonte, May 25^th^, 2023

Dear academic editor and reviewer(s):

With this letter, we would like to thank you for the comments and suggestions which helped revise our work and that certainly perfect and improve our text.

We inform you that the changes suggested by the academic editor and reviewer(s) have been implemented.

Some clarifications are provided below:

As the data allow us to discuss bioethical aspects beyond autonomy, such as beneficence and non-maleficence, we have chosen to change the title in order to make the message clearer and more compatible with the results presented. Therefore, the new title presented is “Bioethical principles in choosing the mode of birth: a retrospective study”.

Regarding the data questioned in Table 1: As these are retrospective data collected from electronic medical records, we were unable to obtain a complete record of all the analyzed variables of the 2507 patients. Thus, the highlighted numbers are the specific N of each evaluated variable. Example: Of the 2507 medical records, 1251 had the maternal age record, 1244 had the type of accommodation, 2253 described the use of analgesia and 2485 had the weight record.

As suggested, we have added the descriptive result of the data presented in Tables 2 and 3.

We await the final answer and are willing to clarify any queries.

Sincerely,

The authors.
