# Supplementary material for: Perinatal complications of the maternal–Fetal dyad in primiparous women subjected to vaginal delivery versus elective cesarean section: A retrospective study of clinical results associated with bioethical precepts
Source: PLoS One. 2023 Oct 17;18(10):e0292846. doi: 10.1371/journal.pone.0292846 (PMC10581477; doi:10.1371/journal.pone.0292846)
Supplement: Attachment — Submitted filename: Comments.docx [file pone.0292846.s005.docx]

The paper addressed the question of whether physicians need to recommend vaginal delivery or cesarean section to their pregnant patients. Based on different parameters, the paper concluded that there are no significant differences between the two procedures that make the physicians recommend one over the other.

The data presented in this paper will add some important information to help the physicians and their patients to understand that there is no difference between these two procedures based on this particular data from a specific ethnic group. I would recommend the publications after considering my below comments.

Comments:

1- It would be interesting to know whether there is a difference between vaginal delivery and cesarean section based on ethnicity (If 2507 women are from different ethnic groups).
2- I am not clear why the authors used different statistical analyses (Mann-Whitney, student’s, and Chi-square to calculate the p values. If there is a reason, the authors need to explain.
3- In all the tables, the authors are confused between the comma (,) and the decimal point (.). For example, when they write 1.515, this is a decimal number that means 1 and 515 fractional. However, 1,515 means a thousand and five hundred fifteen. They need to fix all these comma-period in the three tables (as I fixed in the table below).
4- The authors need to explain what “n” means in all tables, especially if the “n” under each category is less than the total number of deliveries. For example, “General” n = 2,507 but under “Maternal age” n=1,515
5- The numbers inside some parentheses are the percentages, so I added “%” to them (red colors) in the below table as an example. However, I do not know what the other numbers mean. For example, “31.0 (28.0–34.0)”. I yellow-highlighted them in the table.
6- The authors need to define the units in each category. Maternal age (years?), Gestational age (months?), weight (pounds or kilograms?).

Please look at the table below and fix the remaining tables. Please try to keep the tables as simple as you can so the readers can follow them easily without the need to read the text.

| **Characteristic** | **General**  (n=2,507) | **Vaginal delivery**  (n=1,807) | **Cesarean section**  (n=700) | **P value** |
| --- | --- | --- | --- | --- |
| **Maternal age** (years)  (n=1,515) | 30.6 ± 5.0  31.0 (28.0–34.0) | 30.0 ± 4.9  30.0 (27.0–34.0) | 32.1 ± 4.8  32.0 (29.0–35.0) | <0.001^M^ |
| **Accommodation** (n=1,244) |  |  |  | - - |
| Set | 1,244 (100%) | 692 (100%) | 552 (100%) |  |
| ICU | 0 (0.0%) | 0 (0.0%) | 0 (0.0%) |  |
| **Anesthesia**  (n=2,253) |  |  |  | <0.001^Q^ |
| No | 12 (0.5%) | 12 (0.7%) | 0 (0.0%) |  |
| Location | 3 (0.1%) | 3 (0.2%) | 0 (0.0%) |  |
| Epidural | 1,779 (79%) | 1,777 (98.3%) | 2 (0.4%) |  |
| Spinal anesthesia | 459 (20.4%) | 15 (0.8%) | 444 (99.6%) |  |
| **Gestational age** (months) (n=2.498) | 39.4 ± 1.0  39.5 (38.7–40.1) | 39.3 ± 1.0  39.3 (38.6–40.0) | 39.7 ± 0.8  40.0 (39.1–40.1) | <0.001^M^ |
| **Weight (Kg or Ibs?)**  (n=2.485) | 3,213.1 ± 382.5  3,210.0 (2,960.0–3,460.0) | 3,155.8 ± 365.3  3,160.0 (2,920.0–3,405.0) | 3,360.3 ± 386.8  3,350.0 (3.100.0–3,620.0) | <0.001^T^ |
| **PICU** |  |  |  | 0.043^Q^ |
| Yes | 72 (2.9%) | 60 (3.3%) | 12 (1.7%) |  |
| No | 2,435 (97.1%) | 1,747 (96.7%) | 688 (98.3%) |  |
| **5-minute APGAR** |  |  |  | 0.063^Q^ |
| < 7 | 18 (0.7%) | 17 (0.9%) | 1 (0.1%) |  |
| ≥ 7 | 2,489 (99.3%) | 1,790 (99.1%) | 699 (99.9%) |  |
| **Immediate breastfeeding** |  |  |  | <0.001^Q^ |
| Yes | 2,414 (96.3%) | 1,714 (94.9%) | 700 (100%) |  |
| No | 93 (3.7%) | 93 (5.1%) | 0 (0.0%) |  |
| **Maternal death** |  |  |  | - - |
| Yes | 0 (0.0%) | 0 (0.0%) | 0 (0.0%) |  |
| No | 2,507 (100%) | 1,807 (100%) | 700 (100%) |  |
| **Neonatal death** |  |  |  | - - |
| Yes | 0 (0.0%) | 0 (0.0%) | 0 (0.0%) |  |
| No | 2,504 (100%) | 1,804 (100%) | 700 (100%) |  |
| **Maternal readmission** |  |  |  | 0.569^Q^ |
| Yes | 32 (1.3%) | 25 (1.4%) | 7 (1.0%) |  |
| No | 2,475 (98.7%) | 1,782 (98.6%) | 693 (99.0%) |  |
| **Neonatal readmission**  (n=2.499) |  |  |  | <0.001^Q^ |
| Yes | 183 (7.3%) | 177 (9.8%) | 6 (0.9%) |  |
| No | 2,316 (92.7%) | 1,622 (90.2%) | 694 (99.1%) |  |
| **Uterine bleeding** |  |  |  | <0.001^Q^ |
| Usual | 2,222 (88.6%) | 1,574 (87.1%) | 648 (92.6%) |  |
| Increased | 285 (11.4%) | 233 (12.9%) | 52 (7.4%) |  |
| **Transfusion** |  |  |  | >0.999^Q^ |
| Yes | 7 (0.3%) | 5 (0.3%) | 2 (0.3%) |  |
| No | 2,500 (99.7%) | 1,802 (99.7%) | 698 (99.7%) |  |
| **Use of uterotonic** (n=2.506) |  |  |  | <0.001^Q^ |
| Prophylactic | 1,660 (66.2%) | 1,014 (56.1%) | 646 (92.3%) |  |
| Therapeutic | 845 (33.7%) | 792 (43.9%) | 53 (7.6%) |  |
| Puerperal hysterectomy | 1 (0.0%) | 0 (0.0%) | 1 (0.1%) |  |
| **Comorbidities** (n=2.496) |  |  |  | 0.146^Q^ |
| Yes | 564 (22.6%) | 394 (21.8%) | 170 (24.6%) |  |
| No | 1,932 (77.4%) | 1,412 (78.2%) | 520 5.4%) |  |

7- “There was a difference in the mean gestational age between the vaginal delivery group (38 weeks + 6 days to 40 weeks) and the on-demand cesarean section group (39 weeks + 7 days to 40 weeks + 1 day)”.

The parentheses numbers are not clear what do they mean, especially if they are different from table 1? Please explain.

8- Table 2: What is “Neck” means? And the title of the second column is not “statistics”. It can be “Patient number/Percentage” or something similar.
9- There are some minor grammatical and spelling errors, but they can easy be fixed before publication.
