# Supplementary material for: Perinatal complications of the maternal–Fetal dyad in primiparous women subjected to vaginal delivery versus elective cesarean section: A retrospective study of clinical results associated with bioethical precepts
Source: PLoS One. 2023 Oct 17;18(10):e0292846. doi: 10.1371/journal.pone.0292846 (PMC10581477; doi:10.1371/journal.pone.0292846)
Supplement: Attachment — Submitted filename: Response to Reviewers .docx [file pone.0292846.s006.docx]

Belo Horizonte, Aug 08, 2023

Dear academic editor and reviewers:

We would like to thank you for the comments and suggestions that helped in the revision of the study and that certainly improved our text. We incorporated the changes suggested by the academic editor and reviewers in the revised draft.

The following are some clarifications:

- The academic editor's recommendations in review 2 were incorporated into this draft.

- For many years, parturients were coerced into vaginal delivery using the allegation that cesarean section, even elective, meant more risk and higher rates of morbidity and mortality for the maternal–fetal binomial. However, most studies did not exclude emergency cesarean sections or cesarean sections indicated in high-risk pregnancies when comparing outcomes. Our results elucidate the safety of the two modes of delivery, which allows indirect discussion of the right to autonomy in choosing the mode of delivery, in addition to other bioethical principles. Since bioethical principles are not measured or presented in our formal results, we chose to modify the title of the text, the objective, and the conclusion, as suggested by reviewer 4. To avoid generating doubts about the study, we modified the title to: **Perinatal complications of the maternal–fetal dyad in primiparous women subjected to vaginal delivery versus elective cesarean section: A retrospective study of clinical results associated with bioethical precepts**

- Regarding the questions about the data in Table 1:

1- We do not have data on the ethnicities of all the patients because these data are not usually included in the medical records of our hospital, as Brazil is a country with high population miscegenation.
2- Because these retrospective data were collected from electronic medical records, we were unable to fully record all the variables analyzed in the 2507 patients. Thus, the highlighted numbers (N) are the valid numbers of each variable evaluated. For example, of the 2507 medical records, 1251 included the maternal age, 1244 the type of accommodation, 2253 described the use of analgesia, and 2485 the weight. As the meaning of the N value generated doubt, we added this information to the results.
3- Different statistical tests were used according to the characteristics of the variables: the quantitative/numeral variables were evaluated by the Anderson–Darling normality test, which analyzes the distribution pattern. According to its results, the T test was used when the numeral variables presented a normal distribution and the Mann‒Whitney test when they did not present a normal distribution. The chi-squared test was used to compare the qualitative variables.
4- The results of the variables maternal age, gestational age, and weight are presented as medians (first quartile, third quartile). Please see the additions to the legend of Table 1.

All tests were performed by a professional statistician.

- There was a difference in gestational age between the vaginal delivery and elective cesarean section, as most cesarean sections were performed near 39 weeks of gestational age, as required by law, and vaginal delivery is a spontaneous and less predictable event, but this fact did not affect the results or conclusions.

- We changed the title of Column 2 of Table 2 to “Patient number/Percentage”, as suggested.

- The English grammar and usage of the text was revised by American Journal Experts (attached certificate).

- As recommended, two references were added that corroborate the statements of the first paragraph of the Discussion.

We await the final answer and are willing to answer any questions you may have.
