# Supplementary material for: Perinatal complications of the maternal–Fetal dyad in primiparous women subjected to vaginal delivery versus elective cesarean section: A retrospective study of clinical results associated with bioethical precepts
Source: PLoS One. 2023 Oct 17;18(10):e0292846. doi: 10.1371/journal.pone.0292846 (PMC10581477; doi:10.1371/journal.pone.0292846)
Supplement: Attachment — Submitted filename: PlosOne_revision 3.docx [file pone.0292846.s007.docx]

The authors answered all my questions to my satisfaction. I recommend publishing this article in PLOS ONE.

I would recommend one minor change in Table 1, it will make the table crystal clear. The number of patients “n” in the header of each column is misleading because these are the total number of patients; however, in each category, the number of patients is different because they do not have record for each patient in each category, so when I calculate the percentage or the median I get different numbers.

Here is an example:

Accommodation: General (n=2507), Vaginal delivery (n=1807), On-demand (n=700)

(n= 1244)	1244 (100%)	692 (100%)	552 (100%)

If I calculate the percentage based on the header number, I will get a different percentage. For example, vaginal delivery = 692/1807 = 38%, which is not correct. I would suggest getting rid of the headers’ numbers (2507, 1807, and 700) and adding the “n” to each category in the columns.

Example:

Accommodation: General,	Vaginal delivery,	On-demand

n= 1244	n=692	n=552

1244 (100%)	692 (100%)	552 (100%)

By doing so, the reader will easily follow the numbers and calculations.
