# Supplementary material for: Perinatal complications of the maternal–Fetal dyad in primiparous women subjected to vaginal delivery versus elective cesarean section: A retrospective study of clinical results associated with bioethical precepts
Source: PLoS One. 2023 Oct 17;18(10):e0292846. doi: 10.1371/journal.pone.0292846 (PMC10581477; doi:10.1371/journal.pone.0292846)
Supplement: Attachment — Submitted filename: Response to Reviewers .docx [file pone.0292846.s008.docx]

Belo Horizonte, Set 28, 2023

Dear academic editor and reviewers:

We would like to thank you for the comments and suggestions that helped in the revision of the study and that certainly improved our text. We incorporated the changes suggested by the academic editor and reviewers in the revised draft.

The following are some clarifications:

- The reviewer 3 recommendations were incorporated into table 1.

- The limitations of study are added in the conclusion as reviewer 4 suggest.

- References were review, there’s no retracted reference in this article.

We await the final answer and are willing to answer any questions you may have.
